# Nernstian Diagnostics of Imperfect Selectivity in Naphthalene Diimide‐Based Aqueous Organic Redox Flow Battery

**DOI:** 10.1002/advs.74945

**Published:** 2026-03-23

**Authors:** Faudillah Alhumairah, Viktor Gueskine, Tobias Abrahamsson, Ivan Hetman, Frida Domeij, Per Leandersson, Xenofon Strakosas, Cedrik Wiberg, Reverant Crispin, Mikhail Vagin

**Affiliations:** ^1^ Laboratory of Organic Electronics Department of Science and Technology Linköping University Norrköping Sweden; ^2^ Wallenberg Wood Science Center ITN Linköping University Norrköping Sweden; ^3^ Rivus Batteries Göteborg Sweden; ^4^ Unit of Clinical Medicine Occupational and Environmental Medicine Department of Health Medicine and Caring Sciences Linköping University Linköping Sweden; ^5^ Clinical Department of Occupational and Environmental Medicine Region Östergötland Linköping Sweden; ^6^ Wallenberg Initiative Materials Science for Sustainability Department of Science and Technology Linköping University Norrköping Sweden

## Abstract

Aqueous organic redox flow batteries (AORFBs) enhance the sustainability of battery energy storage systems (BESS), supporting a greater integration of renewable electricity into the grid. We investigated the aspartate‐derived naphthalene diimide (NDI)‐based AORFB by independent monitoring of the individual electrode potentials on positive and negative electrodes. The mapping of these electrode potentials against analytically calculated states of charge (SOCs) for the negative and positive electrodes showed good agreement. This manifests the Nernstian behavior of electrode potentials defined by non‐ideal redox electrolytes. Evidence was obtained for both the re‐oxidation of charging products at the negative electrode by oxygen traces and their hydrolysis, which were conceptualized as redox shunt and irreversible degradation, respectively. The contributions of these processes to performance deterioration were illustrated using theoretical charging profiles.

## Graphical abstract

Non‐ideal redox electrolytes still govern the Nernstian behavior of electrode potentials in aqueous organic redox flow batteries under operation. We utilized this effect to analytically predict two scenarios of battery performance degradation.

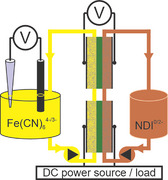

## 1 Introduction

The declining cost of renewable energy and recent geopolitical developments have accelerated the adoption of electricity from renewable sources. However, the intermittent nature of renewable generation poses significant challenges for power grids, arising from temporal and spatial mismatches between supply and demand. Among the different battery energy storage systems (BESS) developed to address grid congestion and intermittent generation, redox flow batteries (RFB) have gained attention as a promising option owing to their scalability, long cycle life, and versatile design that enables the decoupling of power and energy capacity [1]. Water‐soluble organic redox molecules, synthesized from light and earth‐abundant elements, provide a viable pathway for the development of aqueous organic redox flow batteries (AORFB). Recently, naphthalene diimide (NDI)‐based AORFB have attracted significant attention [2, 3, 4, 5, 6, 7, 8] due to the possibility of storing two electrons during redox processes, making them highly appealing for high‐capacity energy storage applications. The modification of the molecular structure of NDI, particularly through altering the side chains, enhances the solubility and energy capacity [9, 10, 11]. The monitoring of the state of charge (SOC) during operation is essential for effective RFB management to avoid degradation. The tracking of the electrical charge passed during charging and discharging, so‐called coulomb counting, is insensitive to the appearance of the possible losses due to the imperfect selectivity of electrode processes [12]. The measurements of open circuit voltage of the entire RFB by zero current interruption of the charging or discharging reflect the overall SOC, but do not account for individual SOCs on negative and positive electrodes [13, 14, 15]. In parallel to physical methods [16], the independent electrochemical measurements on negative and positive electrode systems enable the estimation of individual SOCs [17]. Unlike non‐zero current techniques such as voltammetry, monitoring the open‐circuit potential of individual electrodes is advantageous because it avoids measurement artifacts arising from high concentrations of redox species and electrode material effects.

In this work, we performed in situ zero‐current potentiometric monitoring of electrode potentials as a robust and non‐invasive strategy to independent tracking the individual SOCs of both the negative and positive electrolytes during AORFB operation. This enables the direct comparison between experimental electrode potentials and analytical SOC‐potential relationships derived from the Nernst equations. By applying this method to an AORFB based on aspartate‐derived naphthalene diimide (NDI) and ferrocyanide as the negative and positive redox couples, respectively, we demonstrate that alterations in individual SOC profiles provide direct mechanistic insight into performance deterioration. The in situ SOC monitoring allows us to distinguish between oxygen‐induced charge disbalance and intrinsic chemical degradation of the NDI anolyte, highlighting its utility as a diagnostic tool for understanding attenuation mechanisms in AORFB.

## 2 Experimental Section

### 2.1 Chemicals

All chemicals including ammonium chloride (NH_4_Cl), potassium hexacyanoferrate (II) trihydrate (K_4_Fe(CN)_6_·3H_2_O), potassium ferricyanide (K_3_Fe(CN)_6_), sulfuric acid (H_2_SO_4_, 95%–98%), hydrogen peroxide (H_2_O_2_), sodium hydroxide (NaOH), ammonium hydroxide (NH_4_OH) were purchased from Sigma‐Aldrich and used as received. Nafion‐115 membranes were also purchased from Sigma‐Aldrich. Naphthalene diimide (NDI) was supplied by Rivus Batteries AB (Sweden). NDI was purified before use.

### 2.2 Techniques

#### 2.2.1 NDI Purification

The liquid unpurified sample contained approximately 82% NDI and 10% naphthalene monoimide, with the remaining 8% being dimethyl sulfoxide (DMSO). First, the pH of the unpurified sample was adjusted to pH 3 with sulfuric acid, causing the precipitation of NDI. The precipitate was filtered and dissolved in water using 10% NH_4_OH to adjust pH up to 7. This process was repeated three times to achieve a reproducible ^1^H NMR spectrum (Spinsolve 80 Magritek) of NDI sample diluted tenfold with deionized water.

#### 2.2.2 Membrane Pretreatment

The Nafion membranes were soaked in 3% H_2_O_2_ for 1 h at 80°C to remove organic contaminants and then rinsed with water. The obtained Nafion membranes were soaked in water for 1 h at 80°C, and thereafter in 1% NaOH for 1 h at 80°C [18]. The activated Nafion membranes were stored in water and directly used in RFB assembly.

#### 2.2.3 Electrode pretreatment

Carbon paper AvCarb MGL190 (∼190 um thickness, Fuel Cell Store) was heated in 500°C for 30 min and left overnight before use.

#### 2.2.4 Three‐Electrode Cell Experiments

Solutions either of NDI (50 mm) or K_4_Fe(CN)_6_ (50 mm) in 1 m NH_4_Cl were used in the cyclic voltammetry experiments in a three‐electrode cell using a platinum wire and Ag/AgCl (3 m KCl) as the counter and reference electrodes, respectively. Biologic SP 200 potentiostat was used. The glassy carbon electrode (3 mm) was used as a working electrode.

#### 2.2.5 RFB Experiments

The RFB was assembled by using a commercial single serpentine cell 5SCH (Fuel Cell Technologies Inc.) with geometrical area 5 cm^2^ followed the instructions outlined [19]. PTFE gaskets of 0.4 mm thickness (Nordbergs Tekniska AB, Sweden) were used with a compression rate of 30%. A torque wrench was applied at 7.5 Nm to each bolt. The closed cell sandwiched activated Nafion 115 membrane between three layers of carbon paper on each side. The flow rate of 30 mL min^−1^ was controlled by a peristaltic pump (Hirschmann Rotarus). The galvanostatic charge‐discharge was performed by using a potentiostat (BioLogic SP200) at room temperature. The tanks were filled with 40 mL of a freshly prepared solution of 0.3 m K_4_Fe(CN)_6_ in 1 m NH_4_Cl as posolyte and 20 mL of 0.3 m NDI solution in 1 m NH_4_Cl as negolyte. The NDI tank was bubbled with nitrogen during all tests to prevent oxygen contamination. Capacity utilization was calculated as the ratio of the experimentally obtained discharge capacity to the theoretical capacity. Energy efficiency was determined by numerically integrating the capacity‐voltage profiles of the discharge and charge processes and calculating the ratio of the discharge energy to the charge energy. All numerical integrations were performed using MATLAB's trapz function.

#### 2.2.6 High‐Performance Liquid Chromatography–Mass Spectrometry (HPLC–MS)

NDI purity was analyzed using TSQ Fortis Plus mass spectrometer connected to Vanquish autosampler and pump all from Thermo Scientific. The column was a Syncronis HILIC 150 × 2.1 mm with 5 µm particles (Thermo Scientific). Ten microliter sample was injected, the flow rate was 0.3 mL min^−1^, and the mobile phase consisted of 40% acetonitrile and 60% water. NDI was analyzed with heated electrospray ionization in negative mode (−3000 V), and the precursor and product ions (m/z) were 497.2 and 291.97, respectively. Chromatograms were evaluated with the Software Xcalibur from Thermo Scientific.

NDI degradation products were analyzed using Waters system equipped with a 2767 Sample Manager, dual 515 HPLC pumps, a 2424 Evaporative Light Scattering Detector, a 2998 Photodiode Array detector, and an SQ Detector 2 (single‐quadrupole mass spectrometer, ESI ionization). Separation was carried out using an XBridge BEH C18 column (3.5 µm, 4.6 mm × 50 mm, 130 Å). The mobile phases consisted of (A) water (95%) with acetonitrile (5%) and 10 mm ammonium acetate, and (B) acetonitrile (90%) with water (10%) and 10 mm ammonium acetate. A linear gradient from 10% to 100% B was applied over 6 column volumes, at a flow rate of 1.5 mL/min. NDI samples were diluted to 1 mm, and the injection volume was 20 µL. Data acquisition and processing were performed using MestReNova (v12.0.4).

## 3 Results and Discussion

### 3.1 Half‐Cell

**FIGURE 1:**
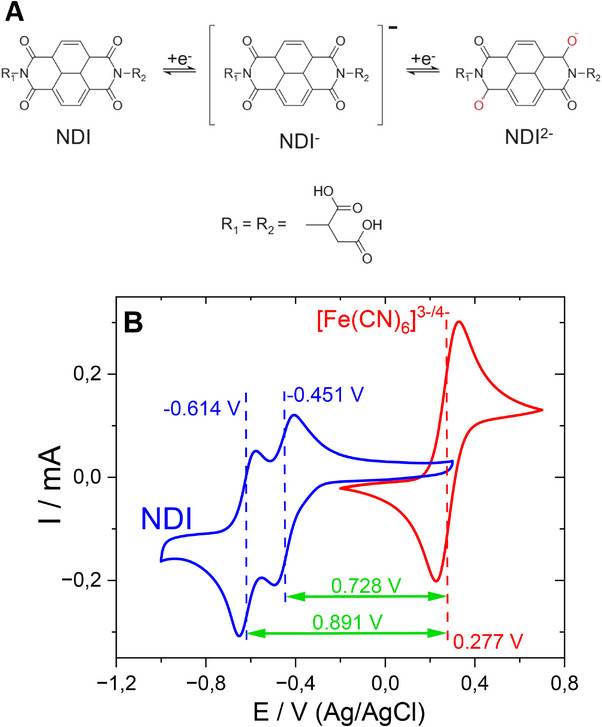
Redox processes (A) and voltammetric response (B) of NDI derivative. The cyclic voltammograms recorded in three‐electrode cell with diluted redox electrolytes (50 mm NDI and 50 mm K_4_(FeCN)_6_, blue and red curves, respectively) in 1 m NH_4_Cl; scan rate 20 mV s^−1^) on glassy carbon electrode.

To increase the concentration of organic redox components, we utilized NDI molecular framework modified at both imide positions with carboxylate‐functionalized alkyl chains, equilibrated by ammonia as counterions (Figure 1A; Note S1). Similarly, ammonia‐based electrolyte was used for the NDI solubility maximization.

The cyclic voltammetry in three‐electrode cell was used to predict the operational cell voltage of AORFB. The high concentrations of redox components utilized in the operational battery as well as high surface area of the activated electrode, define the appearance of high redox currents at the working electrode in three‐electrode cell. In such situations, the recorded voltametric response originates not from working electrode process of interest, but by the processes at the counter electrode (such as oxygen evolution and platinum oxidation). To avoid this, the redox electrolytes were diluted 6 times (from 0.30 m to 0.05 m) prior to voltammetry in the three‐electrode cell.

The voltammetric response of NDI derivative functionalised with ammonium carboxylate side chains (Figure 1A) was recorded on glassy carbon electrode (Figure 1B). Two sequential mono‐electronic redox processes are visible with the half‐peak potentials at −0.451 and −0.614 V (Ag/AgCl). The preservation of reversibility was observed with the increase of the scan rate (Figure S3), illustrating reasonable rate capabilities of the NDI redox conversions. The three‐electrode voltammetry in diluted ferrocyanide solution showed the archetypical reversible mono‐electronic process with half‐peak potential of 0.277 V (Ag/AgCl). The redox peaks of NDI are broader in comparison with the ferrocyanide response, illustrating the diffusional limitations. The differences between the half‐peak potentials of NDI and ferrocyanide allow the estimation of AORFB voltage of 0.728 and 0.891 V sustained by the first and the second mono‐electronic redox processes of NDI.

### 3.2 Full Cell

To compensate the two‐electron redox process of NDI by one‐electron ferrocyanide, the double excess by volume of ferrocyanide solution was fed into the assembled AORFB. The excess of the ammonia‐based background electrolyte (1 m NH_4_Cl) was utilized to assure the high ionic conductivity, which enables the maximization of the electrochemically‐available surface area of porous electrodes. The highest concentration of NDI accessible in such conditions was 0.3 M. The further increase of concentration led to the appearance of the precipitate.

#### 3.2.1 Monitor Cell

**SCHEME 1:**
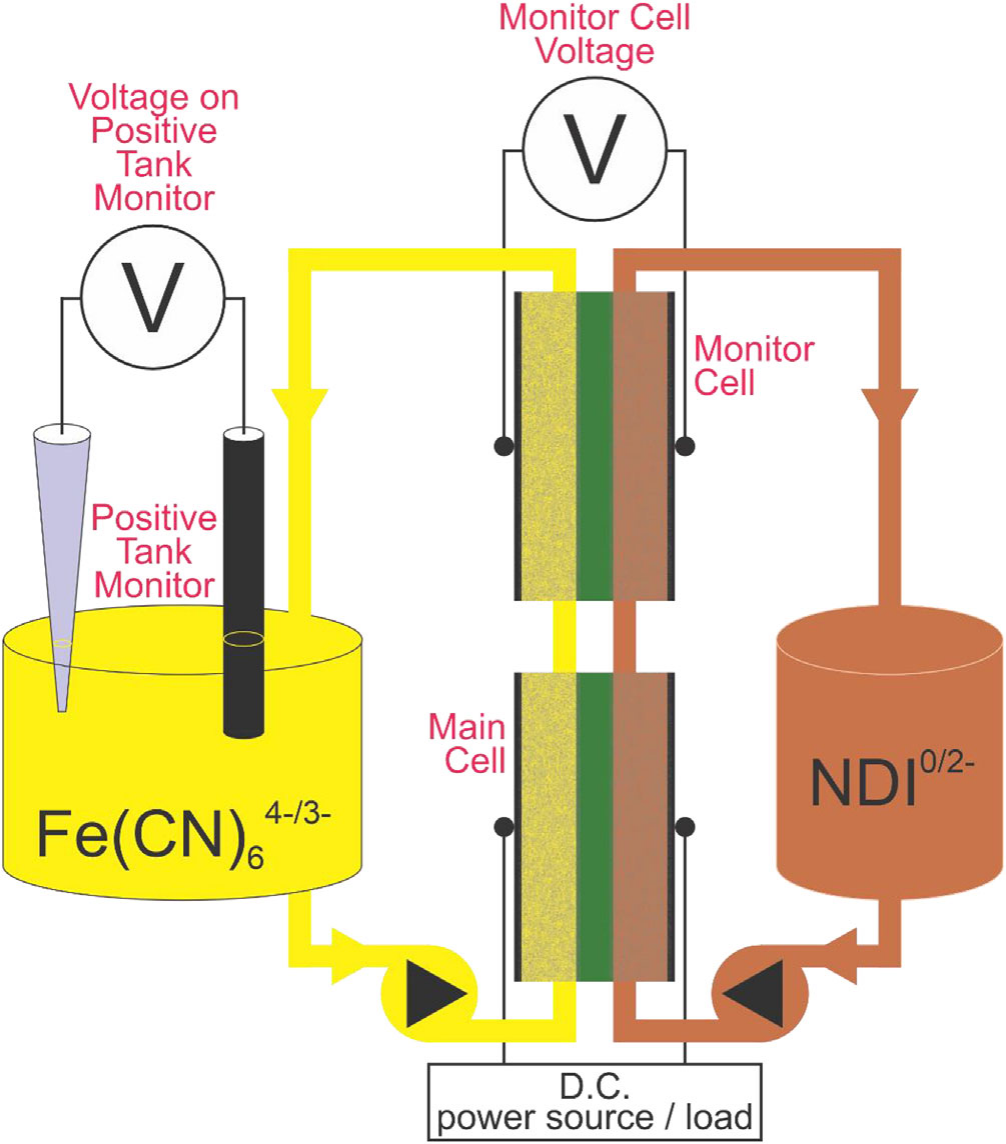
The configuration of AORFB for in situ monitoring of SOCs.

To collect the data for the state of the charge of AORFB in situ, we introduced two additional potential probing cells to the system (Scheme 1). First, the Monitor Cell assembled using the cell fixture identical to the main cell was introduced downstream to the main cell. The addition of such an extra cell increases the hydraulic resistance, leading to higher pumping losses and a reduction in round‐trip efficiency. Therefore, the Monitor Cell should be considered only as a research tool. However, we did not observe any noticeable effect either on the voltage efficiency or on the coulombic efficiency due to the presence of the Monitor Cell.

**FIGURE 2:**
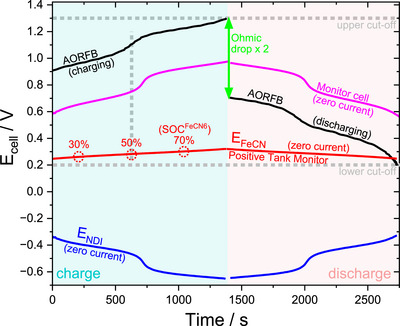
Galvanostatic charge discharge profiles. The time dependencies of experimental potentials on AORFB (under charge and discharge current densities of 100 mA cm^−2^, black curves), Monitor Cell (at the zero current, magenta curves) and Positive Tank Monitor (at the zero current, red curve. The electrode potential on NDI electrode (blue curve) was calculated as the subtraction of Monitor Cell voltage from the voltage on Positive Tank Monitor. The dotted red points illustrate the independent calibration by mixture of Positive Tank Monitor by ferrocyanide/ferricyanide mixtures.

The independent monitoring of the open circuit voltage raised on Monitor Cell due to the different electrode potentials on the negative and positive sides of the device was carried out during AORFB operation. The application of the constant positive current (Figure 2) on AORFB cell results in the coherent growth of the cell voltages on both AORFB cell and the Monitor Cell. The rise of the cell voltage at the zero current conditions (Monitor Cell) illustrates the increase of the difference in potentials on positive and negative electrodes due to the conversion of redox components driven by AORFB cell. This is the battery charging by an external DC power source sustained by the reduction of pristine NDI and the oxidation of ferrocyanide to ferricyanide. The achievement of the upper cut‐off cell voltage triggered the change of the applied current polarity (from 100 to −100 mA cm^−2^), which can initiate the backward conversion of redox components, namely oxidation of reduced NDI and reduction of ferricyanide to ferrocyanide. However, the immediate change of the current at the cut‐off point results in the appearance of the sharp decrease of the cell voltage on AORFB of ca. 0.6 V amplitude. On the contrary, the voltage at the Monitor Cell showed the absence of such decrease. The voltage differences between AORFB cell and the Monitor Cell cut down the operational cell voltages of AORFB limited by the cut‐offs. In other words, the application of currents on AORFB constricts the operational cell voltages by the appearance of the voltage losses. These are the ohmic losses of cell voltage raised due to the finite ohmic resistance of AORFB cell. The 0.6 V ohmic loss raised upon the change of the current from 500 to −500 mA (current density from 100 to −100 mA cm^−2^) can be converted by Ohm's law to the ohmic resistance of 0.6 Ohm (half of the ohmic loss divided by the absolute value of the current).

The application of negative current density started the coherent decrease of cell voltages on both AORFB cell and Monitor Cell, and the backward conversions of redox components (Figure 2). This is the battery discharging on the electrical load. The equality of time spent on charging (e.g., 1378 s at 100 mA cm^−2^) and discharging (e.g., 1324 s at −100 mA cm^−2^) illustrates the efficiency of the electrical energy storage assured by the comparable rates of the forward and backward conversions of AORFB redox components.

Noticeably, both charging and discharging profiles of AORFB cell are non‐monotonous and show the presence of the inflections at ca. 1.11 V and ca. 0.54 V, respectively. These are the transitions between the first and the second mono‐electronic redox process of NDI visualized on three‐electrode cell voltammetry (−0.51–−0.55 V (Ag/AgCl), Figure 1A). The presence of inflection points manifests the access of the second mono‐electronic redox process of NDI in the battery operation. The healthy, well‐balanced AORFB shows the appearance of the inflection point in the middle of the charging or discharging profiles (Figure 2), which corresponds to the point of 50% SOC on positive redox electrolyte.

#### 3.2.2 Positive Tank Monitor

The cell voltage on Monitor Cell is created by the difference in the electrode potentials established on electrodes in NDI and ferro‐/ferricyanide solutions. To decouple them, an independent monitoring of electrode potential in the positive electrolyte during AORFB operation was implemented. For this aim, the tank of the positive electrolyte was equipped with Positive Tank Monitor, which consisted of glassy carbon electrode and Ag/AgCl (3 m KCl) as working and reference electrodes, respectively. The charge and discharge processes driven on AORFB cell resulted in coherent growth and decline of the open circuit voltage recorded on the Positive Tank Monitor. The independent calibration of the Positive Tank Monitor by ferro‐/ferricyanide mixtures of different ratios in identical background electrolyte (Figure S4) enabled the mapping of the potential on Positive Tank Monitor with SOC of positive electrolyte of AORFB. Noticeably, the potential on Positive Tank Monitor created by 50% SOC on positive redox electrolyte shows the full match with the position of the inflection point on the charging profile of AORFB cell. Moreover, the inflection point enables the independent monitoring of the first and the second monoelectronic redox processes of NDI in operational AORFB.

The signal recorded on Positive Tank Monitor (Figure 2) is a potential on the positive electrode of AORFB at zero current conditions (*E_FeCN_*). The potential independently recorded on Monitor Cell is a difference in electrode potentials on positive and negative electrodes at the zero current conditions. Therefore, the subtraction of the potential on Positive Tank Monitor (*E_FeCN_*) from the potential on Monitor Cell gives the estimated potential on negative electrode of AORFB (*E_NDI_*) at the zero current conditions (Figure 2).

### 3.3 Nernstian Determinism

#### 3.3.1 Positive Electrolyte

At any applied potential, the equilibrium electrode potential is established by the redox reaction of ferro‐/ferricyanide

(1) $$Fe{\left(CN\right)}_{6}^{3-}+e^{-}↔Fe{\left(CN\right)}_{6}^{4-}$$

obeys the Nernst equation:

(2) $$E_{FeCN}=E_{FeCN}^{0^{\prime }}+\frac{f}{n}lg\left(\frac{\left[Fe{\left(CN\right)}_{6}^{3-}\right]}{\left[Fe{\left(CN\right)}_{6}^{4-}\right]}\right)$$

where *E_FeCN_* is the potential on the positive electrode of AORFB at zero current conditions (potential measured on Positive Tank Monitor) (V), $E_{FeCN}^{0^{\prime }}$ is the standard electrode potential (V), $\left[Fe{\left(CN\right)}_{6}^{3-}\right]$ and $\left[Fe{\left(CN\right)}_{6}^{4-}\right]$ are the equilibrium concentrations (M) of ferricyanide and ferrocyanide, respectively, *n* is the number of electrons (*n*  =  1 for ferro‐/ferricyanide redox process) and $f=\frac{2.303RT}{F}$(where *R* is the gas constant (8.314 J mol^−1^ K^−1^)), *T* is the absolute temperature (298 K) and *F* is the Faraday's constant (96495 C mol^−1^).

SOC of the each of electrolytes of the redox flow battery is the ratio between the concentration of electrons accommodated by redox process to the total concentration of electronic charge carriers. SOC for positive redox electrolyte ($q_{calculated}^{FeCN}$) is defined as the concentration of electrons expressed as:

(3) $$q_{calculated}^{FeCN}≔\frac{\left[Fe{\left(CN\right)}_{6}^{3-}\right]}{\left[Fe{\left(CN\right)}_{6}^{3-}\right]+\left[Fe{\left(CN\right)}_{6}^{4-}\right]}$$

Using the Nernst equation (Equation (2)), one can derive the analytical equation for the dependence of SOC of positive redox electrolyte on the potential measured on the Positive Tank Monitor (Note S2):

(4) $$q_{calculated}^{FeCN}=\frac{10^{\left(\frac{E_{FeCN}-E_{FeCN}^{0^{\prime }}}{f}\right)}}{\left(1+10^{\left(\frac{E_{FeCN}-E_{FeCN}^{0^{\prime }}}{f}\right)}\right)}$$

#### 3.3.2 Negative Electrolyte

The whole redox process of NDI consists with two individual mono‐electron redox reactions:

(5) $$NDI+e^{-}↔NDI^{-}$$

(6) $$NDI^{-}+e^{-}↔NDI^{2-}$$

where *NDI*, *NDI* ^−^ and *NDI* ^2 −^ are pristine NDI, its radical‐anion and di‐anion, respectively. The overall redox process of NDI is:

(7) $$NDI+2e^{-}↔NDI^{2-}$$

At any applied potential, the equilibrium electrode potential established by redox reactions of NDI obeys the Nernst equation:

(8) $$\begin{array}{ccc} E_{NDI} & = & E_{NDI/NDI^{-}}^{0^{\prime }}+flg\;\left(\frac{\left[NDI\right]}{\left[NDI^{-}\right]}\right)=E_{NDI^{-}/NDI^{2-}}^{0^{\prime }}\\ & & +flg\;\left(\frac{\left[NDI^{-}\right]}{\left[NDI^{2-}\right]}\right)=E_{NDI/NDI^{2-}}^{0^{\prime }}+\frac{f}{2}lg\left(\frac{\left[NDI\right]}{\left[NDI^{2-}\right]}\right) \end{array}$$

where *E_NDI_* is the potential on the negative electrode of AORFB (V) at the zero current conditions, $E_{NDI/NDI^{-}}^{0^{\prime }}$ is the standard electrode potential (V) for Equation (5), $E_{NDI^{-}/NDI^{2-}}^{0^{\prime }}$ is the standard electrode potential (V) for Equation (6), $E_{NDI/NDI^{2-}}^{0^{\prime }}$ is the standard electrode potential (V) for the overall Equation (7), [*NDI*], [*NDI* ^−^] and [*NDI* ^2 −^] are the equilibrium concentrations (M) of *NDI*, *NDI* ^−^ and *NDI* ^2 −^, respectively. Additionally, the standard electrode potential for the overall reaction ($E_{NDI/NDI^{2-}}^{0^{\prime }}$) is defined as:

(9) $$E_{NDI/NDI^{2-}}^{0^{\prime }}=\frac{E_{NDI/NDI^{-}}^{0^{\prime }}+E_{NDI^{-}/NDI^{2-}}^{0^{\prime }}}{2}$$

The SOC on a negative redox electrolyte ($q_{calculated}^{NDI}$) is defined as the ratio of concentration of accommodated electrons to the total concentration of electronic charge carriers:

(10) $$q_{calculated}^{NDI}≔\frac{\left[NDI^{-}\right]+2\left[NDI^{2-}\right]}{\left[NDI\right]+\left[NDI^{-}\right]+\left[NDI^{2-}\right]}$$

Using the Nernst equations (Equation (8)), one can derive the analytical equation of the dependence of SOC of negative redox electrolyte on the potential on negative electrode of AORFB (Note S3):

(11) $$q_{calculated}^{NDI}=\frac{\varepsilon \delta +2}{\varepsilon \delta +1+\varepsilon^{2}}$$

where

(12)

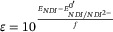

and

(13) $$\delta =10^{\frac{E_{NDI/NDI^{-}}^{0^{\prime }}-E_{NDI^{-}/NDI^{2-}}^{0^{\prime }}}{2f}}$$

To estimate the values of the standard electrode potentials for positive and negative electrodes of AORFB, namely $E_{FeCN}^{0^{\prime }}$ and $E_{NDI/NDI^{2-}}^{0^{\prime }}$, respectively, the smoothed derivative curves of the corresponding time dependence (Figure S5) were used. The position of extremums on such curves defines the values of the standard electrode potential for positive and negative electrodes. In addition, the parameter δ was used as a free parameter of fitting.

The strength of such Nernstian analysis is that the analytical equations (Equations (4) and (11)) enable the prediction of the SOC for any point of the time‐dependent experimental electrode potentials (*E_FeCN_* and *E_NDI_*) during the AORFB operation. Moreover, the whole profile of SOC can be reconstructed using any short part of the curve. Here, the important assumption is the perfect selectivity, namely, the absence of any other redox processes except the main ones, namely, Equation (1) for *E_FeCN_* and Equations (5)–(7) for *E_NDI_*, implies that both the estimated SOCs and actual charge spent on AORFB grow linearly with time.

**FIGURE 3:**
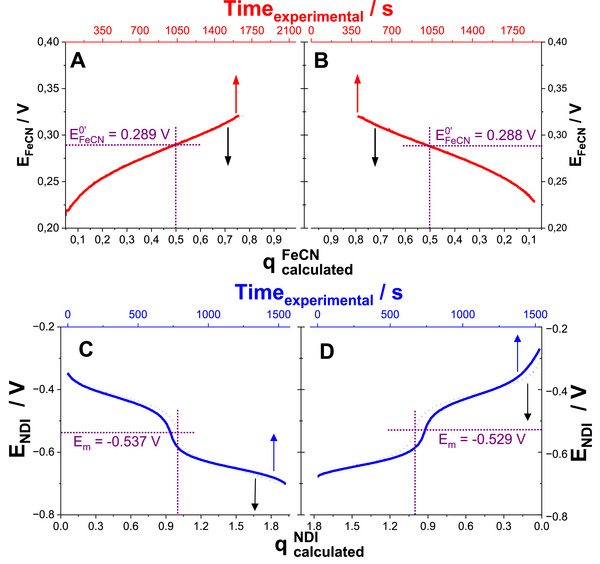
The mapping of time dependencies of electrode potentials by SOC profiles during AORFB operation (100 mA cm^−2^). (A,B) charge and discharge profiles for ferrocyanide/ferricyanide positive electrode, respectively (solid curves – experimental time dependencies of voltage on Positive Tank Monitor; dotted curves—the dependence of SOC calculated using Equation (4) on the potential of ferrocyanide/ferricyanide electrode); (C,D) charge and discharge profiles for NDI negative electrode, respectively (solid curves – time dependencies of negative electrode voltage estimated by the subtraction of Monitor Cell voltage from the voltage on Positive Tank Monitor; dotted curves—the dependence of SOC calculated using Equation (11) on the potential of NDI electrode (δ=100)).

The predicted SOC profiles can be used for the direct mapping of experimental time dependencies of electrode potentials (Figure 3). Noticeably, the experimental profiles of electrode potentials fit well with the theoretical curves. This leads us to an interesting conclusion regarding RFB thermodynamics: the cell voltages and electrode potentials of RFBs exhibit ideal‐solution behavior at the non‐ideal solution conditions. The thermodynamic non‐ideality of concentrated redox electrolytes in RFBs is offset by the ratio of the activities of the reduced and oxidized forms (as described by the Nernst equations, Equations (2) and (8)), since their activity coefficients are approximately equal.

The visual appearance of the time profile of NDI electrode potential showed a full involvement of the redox material in the AORFB operation (Figure 3C,D; Figure S6). This is illustrated by the mirror symmetry of the beginning and the end of the curve as well as the position of inflection point just in the middle of the profile. Strikingly, the mapping of NDI potential profile with its SOC showed incomplete conversion. Regardless of the current density, the maximum reached SOC for NDI ($q_{calculated}^{NDI}$) is only 1.9, while the theoretical limit is 2. On the contrary, the time dependence ferro‐/ferricyanide electrode potential does not fully run down (e.g. at the current density of 100 mA cm^−2^ Figure 3A,B) even in stoichiometry conditions to NDI in AORFB. Such difference in electrode potential behavior indicates the complexity of NDI redox process affected by the possible aggregation.

### 3.4 Nernstian Diagnostics of Performance Deterioration

Imperfect selectivity of the redox processes in AORFB can be due to two possible scenarios on negative electrode reaction:

- Redox shunt: The products of reduction at the negative electrode undergo the re‐oxidation leading to the pristine reagent by reacting directly with unwanted oxidant impurity *X*. In such a scenario, the negative electrode process becomes insufficiently selective for charging (Equations (5) and (6)) due to the losses caused by the redox shunt, similarly to the losses of current through an electric shunt. The additional reactions between the AORFB charging products (*NDI* ^−^ and *NDI* ^2 −^) and the redox shunt are added in parallel to the overall redox process of NDI (Equations (5)–(7)):
(14) $$NDI^{-}+X\to NDI+X_{red}$$
(15) $$NDI^{2-}+2X\to NDI+2X_{red}$$

(16) $$X+e^{-}\to X_{red}$$

where *X* and *X_red_* are the pristine and reduced forms of the redox shunt. The overall process to drive the non‐selective additional currents thought the redox shunt can be represented as:

Importantly, the NDI components are in big excess with respect to the concentration of *X*. In such conditions, the reactions of redox shunt (Equations (14) and (15)) assures the quick disappearance of *X* via Equation (16). In such scenario the charging process on negative electrode (Equations (5) and (6)) has an additional source of electrons from reduction of *X* (Equations (14) and (15)). This results in the creation of excess in electrical charge on negative electrode system with respect to positive one, which is charge imbalance. Importantly, both analytical equations of the SOCs of negative and positive redox electrolytes (Equations (4) and (11)) remain intact. This is due to the product of additional reactions (Equations (14) and (15)) is pristine NDI available for redox conversion (Equations (5) and (6)).

**FIGURE 4:**
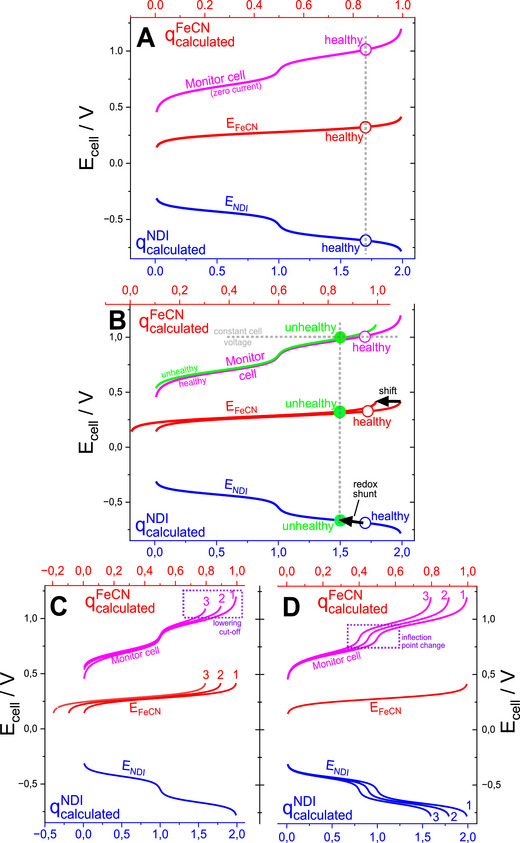
The appearance of AORFB disbalances. The calculated dependencies of SOC for positive and negative electrodes (red curves (using Equation (4)) and blue curves (using Equation (11)), respectively) on electrode potential. The dependencies of Monitor Cell potential (magenta and green curves) are reconstructed by the sum of absolute values of positive and negative electrode potentials. (A) Healthy well‐balanced AORFB; (B) appearance of the charge disbalance due to the redox shunt on negative electrode resulted in positive electrode limitation; (C) effect of the increase of the charge disbalance by redox shunt (α_*redox* *shunt*_ = 0.1); (D) redox disbalance by degradation (β_*irreversible*_ = 0.9).

The healthy well‐balanced AORFB (Figure 4A) is featured with balanced negative and positive electrode systems, which implies that at the any point of time:

(17) $$\;q_{calculated}^{FeCN}=q_{calculated}^{NDI}$$

The appearance of redox shunt during charging (Figure 4B) leads to a decrease of the SOC for NDI ($q_{calculated}^{NDI}$) and the coherent decrease of electrode potential for NDI (*E_NDI_*; Figure 4B, ‘unhealthy’ point on NDI profile) with respect to the healthy well‐balanced AORFB. In parallel, the positive electrode system remains intact, which implies that AORFB upon charging started to be unbalanced due to extra charge available for the negative electrode and limited by the positive electrode:

(18) $$\;q_{calculated}^{FeCN}=q_{calculated}^{NDI}+\alpha_{redox\;shunt}$$

where α_*redox* *shunt*_ is the additional SOC appeared on the negative electrode system due to the redox shunt (0 < α_*redox* *shunt*_ < 1).

The cell voltage must visualize such charge disbalance. To represent the cell voltage appeared on the Monitor Cell of disbalanced AORFB, the linear shift of the intact profile for ferrocyanide/ferricyanide electrode in the direction of the higher SOC (higher $q_{calculated}^{FeCN}$) is used (Figure 4B, ‘unhealthy’ point on FeCN profile). The continuous charge/discharge cycling on AORFB leads to the proliferation of imbalance due to redox shunt.

Importantly, as soon as the redox shunt does not cause irreversible loss of NDI, then the negative electrode system needs neither replacement nor replenishment. The battery can be healed by negolyte re‐balancing.

B. Irreversible degradation: The products of reduction at the negative electrode undergo the attack leading to products, which are redox‐inactive in a time domain of the experiment. The additional reactions are added in parallel to the overall redox process of NDI (Equations (5)–(7)):
(19) $$NDI^{2-}+Y\to Z1$$
(20) $$NDI^{-}+Y\to Z2$$

(21) $$\;q_{calculated}^{FeCN}=q_{calculated}^{NDI}\times \beta_{irreversible}$$

where *Y*, *Z*2 and *Z*2 are the new reagent and the products of different stoichiometries of the new reactions of reduced forms of NDI, respectively. Here, as the redox material is continuously consumed and removed from the equilibria, which defines the electrode potential on NDI (Equations (5)–(7)). In contrast to the case (A), the right part of equation of SOC profile of NDI (Equation (10)), namely $\frac{\left[NDI^{-}\right]+2\left[NDI^{2-}\right]}{\left[NDI\right]+\left[NDI^{-}\right]+\left[NDI^{2-}\right]}$, is affected. The SOC profile for NDI compresses because of irreversible degradation (case (B)): where β_*irreversible*_ is the irreversible loss coefficient (0 < β_*irreversible*_ < 1).

The profiles of cell voltage of AORFB during galvanostatic charging or discharging can be reconstructed by the addition or subtraction of ohmic drop to or from the corresponding potential profiles on the Monitor Cell, respectively (Figure 2). The voltage on Monitor Cell is the sum of the absolute values of the negative and positive electrode potentials (*E_NDI_* and *E_FeCN_*, respectively; Figure 2). The voltage profiles of the Monitor Cell during AORFB charging under redox shunt (Figure 4C) or degradation (Figure 4D) showed a clear difference. In contrast to profiles obtained for AORFB with irreversible degradation, the profiles for the AORFB with redox shunt (Equations (14) and (15) showed a decrease in the cut‐off voltage. The position of the inflection point changes for AORFB with irreversible degradation, while it remained intact for the redox shunt.

### 3.5 Performance

**FIGURE 5:**
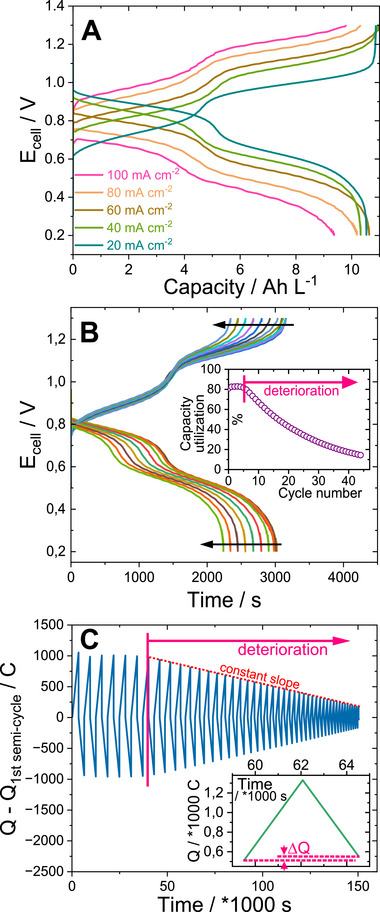
Performance characteristics. (A) the dependence of charge/discharge capacity on the cell voltage for different current densities; (B) continuous cycling of AORFB at 60 mA cm^−2^ (Inset: the AORFB performance deterioration visible in decrease of capacity utilization on the cycle number); (C) the dependence of the background‐subtracted charge on the time of cycling at 60 mA cm^−2^ (background is a charge on the first charge‐discharge semi‐cycle; Inset: the 11th cycle illustrating the charge disbalance on AORFB).

The increase in charge‐discharge current density led to a decrease of the electrical charge stored and released (Figure 5A). In parallel to high coulombic efficiency (CE, the ratio between times spent on discharge and charge; 98% and 94% for 20 mA cm^−^^2^ and 100 mA cm^−^^2^ charge‐discharge current densities, respectively), both energy efficiency (EE, the ratio between the integrated areas of discharge and charge at Figure 5A) and voltage efficiency (VE, EE divided by CE) drop sharply (EE: from 80% to 45% for current densities of 20 mA cm^−^^2^ and 100 mA cm^−^^2^, respectively; VE: from 82% to 47% for current densities of 20 mA cm^−^^2^ and 100 mA cm^−^^2^, respectively).

Noticeably, the charge discharge profiles recorded at the lowest current density (20 mA cm^−^^2^) are featured with the sharp breakdowns of cell voltage, which could represent the predicted decrease of the cut‐off due to the appeared charge disbalance (Figure 4C).

All NDI‐based AORFB showed the performance deterioration during continuous galvanostatic charge‐discharge cycling (Table S1). In our system, the completion of 45 charge‐discharge cycles at the current density of 60 mA cm^−^^2^ resulted in the decrease of both EE (from 65% to 44%) and VE (from 68% to 50%, Figure S7). The ratio between the experimental value of capacity and the theoretical one, namely capacity utilization, faded from 95% down to 20% as a result of cycling (Inset of Figure 5B).

Decisively, the re‐filling of positive tank of discharged deteriorated AORFB by balanced ferrocyanide solution (SOC = 0) results in the performance recovery (Figure S8). The high stability of aqueous ferrocyanide/ferricyanide solutions excludes its degradation. Therefore, the AORFB performance recovery by re‐filling illustrates the intactness of both NDI and its SOC profile equation (Equation (11)) and just a re‐balancing of the positive electrode system.

The only candidate to deteriorate the performance via redox shunt (reagent *X* in Equations (14) and (15)) is the oxygen contamination, which results in quick bi‐electronic oxygen reduction reaction on reduced NDI species yielding hydrogen peroxide:

(22) $$2NDI^{-}+O_{2}+2H_{3}O^{+}\to 2NDI+H_{2}O_{2}+2H_{2}O$$

(23) $$NDI^{2-}+O_{2}+2H_{3}O^{+}\to NDI+H_{2}O_{2}+2H_{2}O$$

The protection of the negative tank by the constant nitrogen flow did not help to improve the stability of AORFB, as soon as the performance deterioration is clearly visible on AORFB. The direct measurements by the oxygen sensor showed the absence of free oxygen in the head space of the negative tank. Because the amount of NDI reduction products significantly exceeds the dissolved oxygen concentration, and because the bulk conversion (Equations (22) and (23)) is fast, the oxygen concentration in the negative electrolyte does not remain constant and becomes negligible at high SOC. On the contrast, the presence of hydrogen peroxide as a product of the oxygen reduction reaction on reduced forms of NDI was qualitatively shown (Note S4).

Considering that the negative tank headspace was under a constant flow of nitrogen and given the large cell walls and gaskets of the AIRFB system, one can deduce that the most likely source of oxygen leakage was through the plastic tubing, which remained exposed to air. The rate of oxygen leakage from the environment into the AORFB is defined as an area of leakage (tubing surface) in contact with NDI normalized by the volume of the NDI solution. This implies that AORFB equipped with the larger tanks experiences slower rate of oxygen contamination and higher stability. Here, the scaling‐up mitigates instability.

The time dependencies of background‐subtracted electrical charge consumed and released by AORFB galvanostatic charging and discharging, respectively, is another tool to investigate AORFB performance (Figure 5C). The parallelism of all ascending lines as well as of all descending lines in each individual cycle represents the charging and discharging by the constant current, namely galvanostatic operation of AORFB. The cycles from first to seventh are close to identical, which is visible by the parallelism of time‐dependencies for lower and higher limits of charge. This is coherent with the dynamics of capacity utilization (Inset in Figure 5B) and of EE and VE (Figure S7), where the low cycles show the good stability. However, after ca. eighth cycle the charge accommodated and released started to decrease, which is visible as shrinkage of the time spent upon each cycle. This is performance deterioration. The charge disbalance is clearly seen in each individual cycle (e.g. 11th cycle, Inset in Figure 5C). The charge accommodated during the AORFB charging (ascending part of the charge‐discharge triangle) is larger than the charge released during the discharging (descending part of the triangle). Decisively, the deterioration is featured with constant slope on time dependence of the charge lower limit. We believe that such confinement of the slope without any visible bending could illustrate the independence of the deterioration rate on the redox components concentration, namely ‘zero order’ process, which manifests the big excess of the NDI with respect to oxygen.

Our system showed the performance characteristics comparable with the reported state‐of‐the‐art (Table S1) [10, 20, 21, 22, 23]. The observed low cycling stability relative to some reported NDI‐based AORFB [24] can be explained mainly by the oxygen effect. The published reports on cycling stability employ an advanced de‐oxygenation infrastructure [2, 5], while we used on‐table de‐oxygenation solely by bubbling nitrogen through the negative tank, while the plastic tubing—the main source of oxygen leakage—remained exposed to air. Additionally, the present system was cycled at a higher current density than in many literature reports, which, in parallel to raised losses [25, 26, 27], could provoke the degradation of redox materials the porous electrode.

**FIGURE 6:**
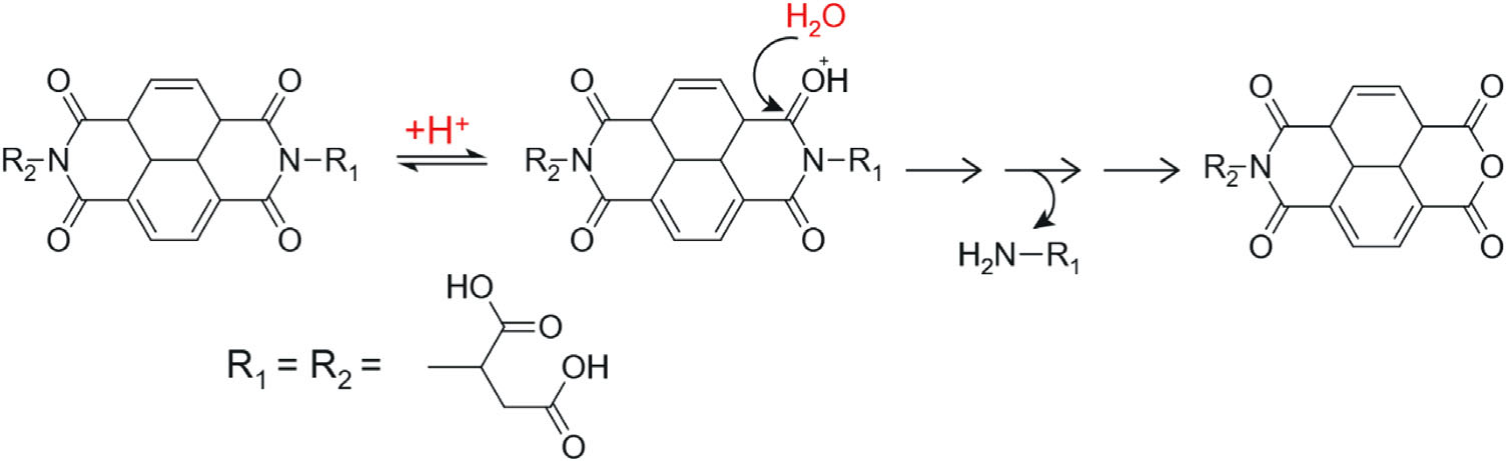
Acidic hydrolysis on NDI by Gabriel synthesis of primary amines.

The voltammetry on diluted NDI‐based electrolyte acquired before and after AORFB operation qualitatively showed the decrease of the redox currents (Figure S9). We evaluated molecular structures in the negative electrolyte before and after AORFB operation by HPLC‐MS (Note S5 and Figures S9–S16). In parallel to pristine NDI, the postmortem sample showed the presence of two new compounds. The operation of AORFB resulted in an increase in the content of monoimide NDI as well as the appearance of aspartate. This illustrates the degradation of NDI via acidic hydrolysis by Gabriel synthesis of primary amines (Figure 6; Note S6). As soon as these reactions are catalyzed by both protons and hydroxide anions, the highest stability of NDI could be expected in neutral media. The visible degradation of NDI during the operation of neutral AORFB could be due to four reasons.

1. The negative charge of AORFB charging products, namely *NDI* ^−^ and *NDI* ^2 −^, facilitates the protonation as the starting position of degradation.
2. Alkyl sulfonate of Nafion membrane has a distinct reactivity as a nucleophile [28], which could contribute to hydrolysis.
3. The contamination of NDI solution with iron species (Figure S17) due to crossover could aggravate the hydrolysis rate. The possible complex formation between iron species and ionic groups of NDI enhances the leaving group ability.
4. The porous negative electrode could experience localized pH changes with respect to the pH in the bulk of negative electrolyte. The protonation of *NDI* ^2 −^ below 3.95 [29], which is quite close to the pH in AORFB (pH 6 and pH 7 before and after AORFB operation), enables the transformation of Equation (6) to proton‐coupled electron transfer:
(24) $$NDI^{-}+e^{-}+H^{+}↔H-NDI^{-}$$

At the conditions of low buffer capacity of ammonia chloride (*pK_a_* of ammonia is 9.2–9.3), the proceeding of redox Equation (24) from left to right (reduction) and from right to left (oxidation) on a highly porous electrode could result in the appearance of localized alkalization and acidification, respectively [30], which could increase the hydrolysis rate.

Importantly, the presence of both hydrogen peroxide and NDI monoimide in the negative electrode system of AORFB manifests the co‐existence of both redox shunt by oxygen reduction reaction (Equations (22) and (23)) and irreversible degradation by hydrolysis (Equations (19) and (20)), respectively:

(25) $$\;q_{calculated}^{FeCN}=q_{calculated}^{NDI}\times \beta_{irreversible}+\alpha_{redox\;shunt}$$

The demarcation of contributions from two processes might be possible via real‐time processing of experimental charge discharge profiles.

## 4 Conclusions

The in situ monitoring of the SOC of AORFB enabled the deconvolution of voltages on positive and negative electrodes. The experimental dependencies of SOCs on individual electrode potentials obey Nernstian analytical equations, implying the achievement of redox equilibria at any time of device operation. The imperfect selectivity of the negative redox process leads to disbalance of the AORFB and performance deterioration. We propose two scenarios for deterioration, namely redox shunt re‐oxidizing negative redox electrolyte and irreversible degradation. The dependencies of the SOC on the cell voltage predicted using Nernstian analytical equations showed the difference between the two scenarios of deterioration. The postmortem analysis of the negative redox electrolyte showed the presence of both hydrogen peroxide and the products of NDI hydrolysis. This illustrates the co‐existence of both redox shunt by oxygen traces and irreversible degradation.

## Conflicts of Interest

The authors declare no conflicts of interest.

## Supporting information




**Supporting File**: advs74945‐sup‐0001‐SuppMat.docx.

## Data Availability

The data that support the findings of this study are available from the corresponding author upon reasonable request.
