# Supplementary material for: Nernstian Diagnostics of Imperfect Selectivity in Naphthalene Diimide‐Based Aqueous Organic Redox Flow Battery
Source: Adv Sci (Weinh). 2026 Mar 23;13(32):e74945. doi: 10.1002/advs.74945 (PMC13252606; doi:10.1002/advs.74945)
Supplement: Supplementary file 1 — Supporting File: advs74945‐sup‐0001‐SuppMat.docx. [file ADVS-13-e74945-s001.docx]

**SUPPORTING INFORMATION**

**Nernstian diagnostics of imperfect selectivity in naphthalene diimide-based aqueous organic redox flow battery**

Faudillah Alhumairah,^1,2^ Viktor Gueskine,^1,2,3^ Tobias Abrahamsson,^1^ Ivan Hetman,^1,4,5^ Frida Domeij,^4,5^ Per Leanderson,^4,5^ Xenofon Strakosas,^1^ Cedrik Wiberg,^3^ Reverant Crispin,^1,2,6^ Mikhail Vagin^1,6^*

^1^Laboratory of Organic Electronics, Department of Science and Technology, Linköping University, 60174 Norrköping, Sweden

^2^Wallenberg Wood Science Center, ITN, Linköping University, 60174 Norrköping, Sweden

^3^Rivus Batteries, Medicinaregatan 8B, 41390 Göteborg, Sweden

^4^Unit of Clinical Medicine, Occupational and Environmental Medicine, Department of Health, Medicine and Caring Sciences, Linköping University, 58183 Linköping, Sweden

^5^Clinical Department of Occupational and Environmental Medicine, Region Östergötland, 58185 Linköping, Sweden

^6^Wallenberg Initiative Materials Science for Sustainability, Department of Science and Technology, Linköping University, 60174 Norrköping, Sweden

*Corresponding author: +46702753087, mikhail.vagin@liu.se

**Table of Contents**

**Figures / Tables / Notes Page**

1. **Supporting Note 1** 3
2. **Figure S1** 3
3. **Figure S2** 4
4. **Figure S3** 5
5. **Figure S4** 6
6. **Supporting Note 2** 7
7. **Supporting Note 3** 8
8. **Figure S5** 10
9. **Figure S6** 11
10. **Figure S7** 12
11. **Table S1** 13
12. **Figure S8** 17
13. **Supporting Note 4** 18
14. **Figure S9** 19
15. **Supporting Note 5** 20
16. **Figure S10** 21
17. **Figure S11** 21
18. **Figure S12** 22
19. **Figure S13** 23
20. **Figure S14** 24
21. **Figure S15** 24
22. **Figure S16** 25
23. **Figure S17** 26
24. **Supporting Note 6** 27

**Supporting Note 1.**

**Characterization of NDI.**

The purified NDI was characterized by ^1^H NMR (Spinsolve 80 [Magritek](https://magritek.com/products/benchtop-nmr-spectrometer-spinsolve/spinsolve-80/)) and liquid chromatography combined with mass spectroscopy. In ^1^H NMR measurements the solution of NDI was diluted with water 1:10. δ _H_ (80 MHz) 8.68 (2 H, s), 5.85 (1 H, dd, *J* 8.7, 6.0), 2.98 (2 H, qd, *J* 14.8, 7.4). The purification on NDI lead is illustrated by the disappearance of high intensity peak at 2.5 ppm associated with DMSO. The presence of hydrogen atoms covalently attached to naphthalene is visible as the peak at ca. 8.7 ppm. The presence of hydrogen atoms associated with aspartate is visible by the presence of the grouped signals at 3.0 ppm and ca. 5.8 ppm.


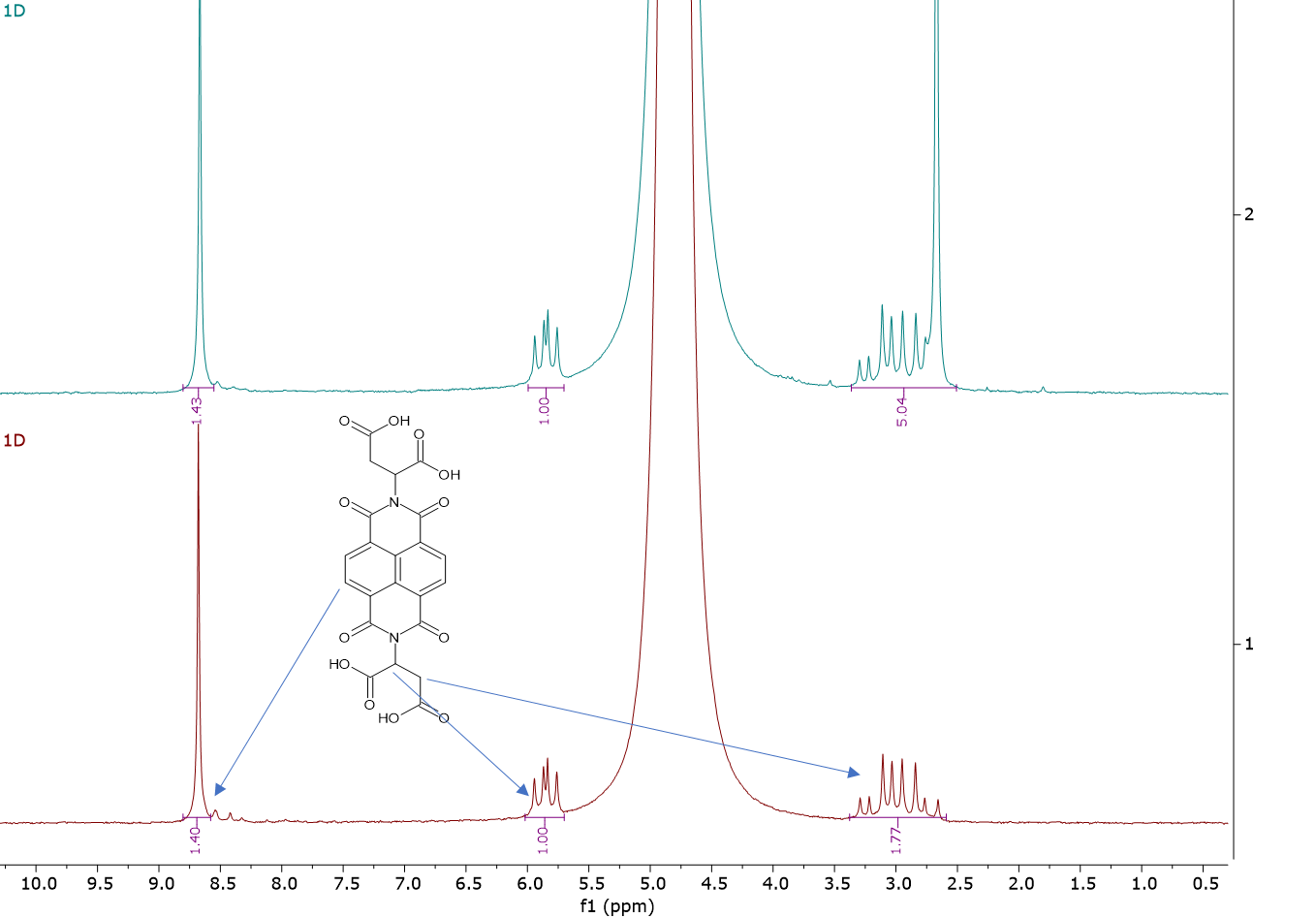


**Figure S1. ^1^**H NMR before and after purification (blue and red curves, respectively).

For LC-MS analysis, NDI solution was diluted to 0.5 µmol L^-1^ by 40% acetonitrile. PFP column (reversed phase C18-column) was used. The injected volume was 10 µL, while the flow rate was 300 µl min^-1^. The negative ionization mode was used for MS with the flow rate of the syringe pump of 5 µl min^-1^. The NDI signals at m/z 497.14 and 498.19 are clearly seen illustrating high purity of the sample.


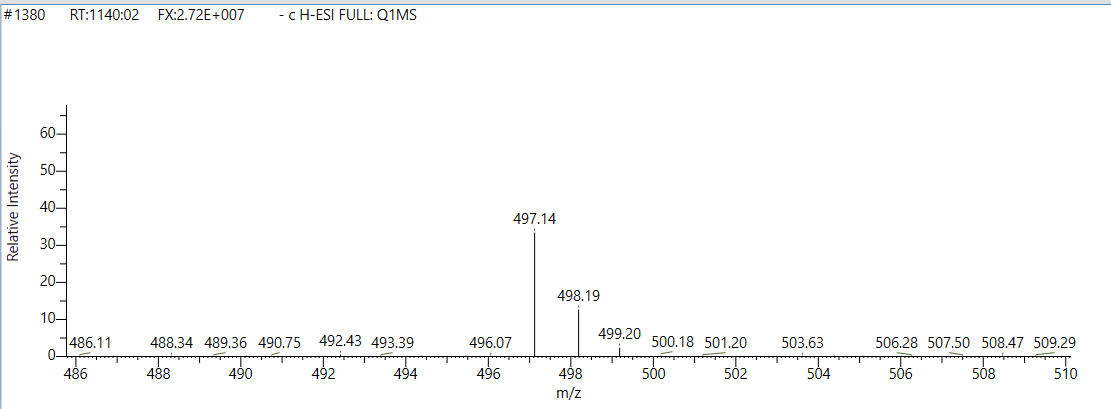


**Figure S2.** LCMS Chromatogram of NDI solution.


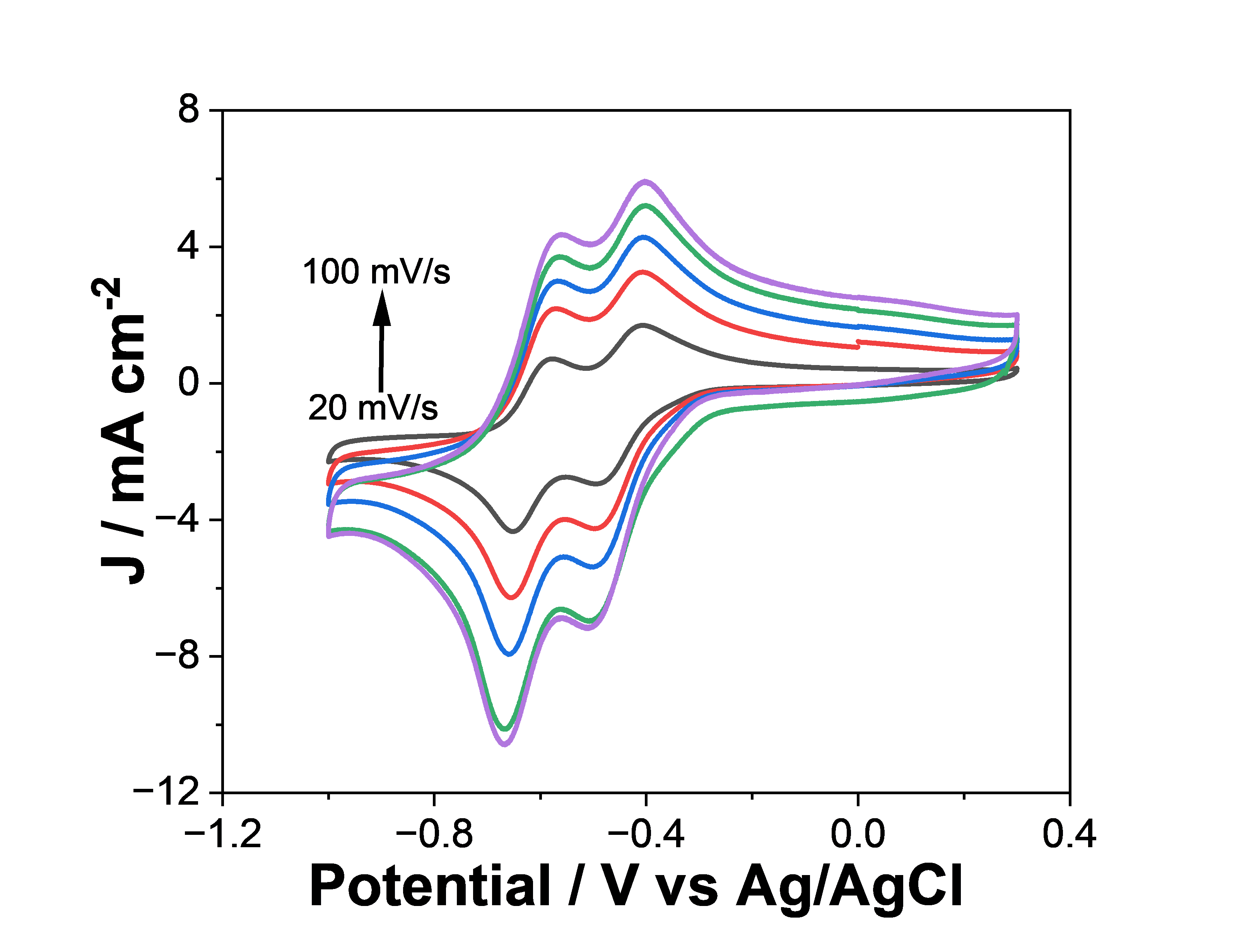


**Figure S3.** Voltammetry of diluted NDI solution (50 mM in 1 M NH_4_Cl) in three-electrode cell at different scan rates.

**Figure S4.** Calibration of open circuit potential on Positive Tank Monitor by the SOC of ferricyanide/ferrocyanide (1M NH_4_Cl, total concentration of iron 0.3 M).

**Supporting Note 2.**

**The equation for SOC on positive electrode**

$q_{calculated}^{FeCN}:=\frac{\left[ Fe\left( CN \right)_{6}^{3-} \right]}{\left[ Fe\left( CN \right)_{6}^{3-} \right]+\left[ Fe\left( CN \right)_{6}^{4-} \right]}$

then

$1-q_{calculated}^{FeCN}=1-\frac{\left[ Fe\left( CN \right)_{6}^{3-} \right]}{\left[ Fe\left( CN \right)_{6}^{3-} \right]+\left[ Fe\left( CN \right)_{6}^{4-} \right]}=\frac{\left[ Fe\left( CN \right)_{6}^{3-} \right]+\left[ Fe\left( CN \right)_{6}^{4-} \right]-\left[ Fe\left( CN \right)_{6}^{3-} \right]}{\left[ Fe\left( CN \right)_{6}^{3-} \right]+\left[ Fe\left( CN \right)_{6}^{4-} \right]}=\frac{\left[ Fe\left( CN \right)_{6}^{3-} \right]}{\left[ Fe\left( CN \right)_{6}^{3-} \right]+\left[ Fe\left( CN \right)_{6}^{4-} \right]}$

And then $\frac{q_{calculated}^{FeCN}}{1-q_{calculated}^{FeCN}}=\frac{\left[ Fe\left( CN \right)_{6}^{3-} \right]}{\left[ Fe\left( CN \right)_{6}^{4-} \right]}$

Update the Nernst equation for ferro-/ferricyanide redox reaction (Eq. (2)) gives:

$E_{FeCN}=E_{FeCN}^{0'}+flg\left( \frac{q_{calculated}^{FeCN}}{1-q_{calculated}^{FeCN}} \right)$

$\frac{q_{calculated}^{FeCN}}{1-q_{calculated}^{FeCN}}={10}^{\left( \frac{E_{FeCN}-E_{FeCN}^{0'}}{f} \right)}$

$q_{calculated}^{FeCN}={10}^{\left( \frac{E_{FeCN}-E_{FeCN}^{0'}}{f} \right)}-q_{calculated}^{FeCN}{10}^{\left( \frac{E_{FeCN}-E_{FeCN}^{0'}}{f} \right)}$

$q_{calculated}^{FeCN}\left( 1+{10}^{\left( \frac{E_{FeCN}-E_{FeCN}^{0'}}{f} \right)} \right)={10}^{\left( \frac{E_{FeCN}-E_{FeCN}^{0'}}{f} \right)}$

and finally, $q_{calculated}^{FeCN}=\frac{{10}^{\left( \frac{E_{FeCN}-E_{FeCN}^{0'}}{f} \right)}}{\left( 1+{10}^{\left( \frac{E_{FeCN}-E_{FeCN}^{0'}}{f} \right)} \right)}$

**Supporting Note 3.**

**The equation for SOC on negative electrode**

The mole fractions of all redox components of NDI system are:

$X_{NDI}=\frac{\left[ NDI \right]}{\left[ NDI \right]+\left[ {NDI}^{-} \right]+\left[ {NDI}^{2-} \right]}$

$X_{{NDI}^{-}}=\frac{\left[ {NDI}^{-} \right]}{\left[ NDI \right]+\left[ {NDI}^{-} \right]+\left[ {NDI}^{2-} \right]}$

$X_{{NDI}^{2-}}=\frac{\left[ {NDI}^{2-} \right]}{\left[ NDI \right]+\left[ {NDI}^{-} \right]+\left[ {NDI}^{2-} \right]}$

Using the Nernst equation for NDI (Eq. (8)), we can write:

$\frac{X_{NDI}}{X_{{NDI}^{-}}}=\frac{\left[ NDI \right]}{\left[ {NDI}^{-} \right]}={10}^{\frac{E_{NDI}-E_{NDI/NDI^{-}}^{0'}}{f}}$ (S1)

$\frac{X_{{NDI}^{-}}}{X_{{NDI}^{2-}}}=\frac{\left[ {NDI}^{-} \right]}{\left[ {NDI}^{2-} \right]}={10}^{\frac{E_{NDI}-E_{NDI^{-}/NDI^{2-}}^{0'}}{f}}$ (S2)

The standard electrode potential for the overall reaction ($E_{NDI/NDI^{2-}}^{0'}$) is the mean value of the standard potentials of individual mono-electronic steps:

$E_{NDI/NDI^{2-}}^{0'}=\frac{E_{NDI/NDI^{-}}^{0'}+E_{NDI^{-}/NDI^{2-}}^{0'}}{2}$

The half of the difference between standard potentials of individual mono-electronic steps:

$\Delta E^{0'}=\frac{E_{NDI/NDI^{-}}^{0'}-E_{NDI^{-}/NDI^{2-}}^{0'}}{2}$

Then,

$E_{NDI/NDI^{2-}}^{0'}+ \Delta E^{0'}=E_{NDI/NDI^{-}}^{0'}$

And

$E_{NDI/NDI^{2-}}^{0'}- \Delta E^{0'}=E_{NDI^{-}/NDI^{2-}}^{0'}$

Then we can rewrite Eq. (S1) as:

$\frac{X_{NDI}}{X_{{NDI}^{-}}}=\frac{\left[ NDI \right]}{\left[ {NDI}^{-} \right]}={10}^{\frac{E_{NDI}-E_{NDI/NDI^{-}}^{0'}}{f}}={10}^{\frac{E_{NDI}-E_{NDI/NDI^{2-}}^{0'}- \Delta E^{0'}}{f}}$ (S3)

and Eq. (S2) as:

$\frac{X_{{NDI}^{-}}}{X_{{NDI}^{2-}}}=\frac{\left[ {NDI}^{-} \right]}{\left[ {NDI}^{2-} \right]}={10}^{\frac{E_{NDI}-E_{NDI^{-}/NDI^{2-}}^{0'}}{f}}={10}^{\frac{E_{NDI}-E_{NDI/NDI^{2-}}^{0'}+ \Delta E^{0'}}{f}}$ (S4)

Let’s assign $\varepsilon\equiv{10}^{\frac{E_{NDI}-E_{NDI/NDI^{2-}}^{0'}}{f}}$, and $\delta\equiv{10}^{\frac{\Delta E^{0'}}{f}}$

Then Eq. (S3) can be written as:

$\frac{X_{NDI}}{X_{{NDI}^{-}}}=\frac{\varepsilon}{\delta}$ (S5)

and

$\frac{X_{{NDI}^{-}}}{X_{{NDI}^{2-}}}=\varepsilon\delta$ (S6)

and

$X_{{NDI}^{-}}=\varepsilon\delta X_{{NDI}^{2-}}$ (S7)

The mass balance for NDI redox reactions can be written as:

$X_{NDI}+X_{{NDI}^{-}}+X_{{NDI}^{2-}}=1$

Which can be rearranged using Eq. (S6) as:

$X_{NDI}=1-X_{{NDI}^{-}}-X_{{NDI}^{2-}}=1-\varepsilon\delta X_{{NDI}^{2-}}-X_{{NDI}^{2-}}$ (S8)

Updating of Eq. (S5) with Eq. (S7) and Eq. (S8) gives:

$\frac{X_{NDI}}{X_{{NDI}^{-}}}=\frac{1-\varepsilon\delta X_{{NDI}^{2-}}-X_{{NDI}^{2-}}}{\varepsilon\delta X_{{NDI}^{2-}}}=\frac{\varepsilon}{\delta}$

Rearrangement gives: $1-X_{{NDI}^{2-}}\left( \varepsilon\delta+1+\varepsilon^{2} \right)=0$

and also: $X_{{NDI}^{2-}}=\frac{1}{\varepsilon\delta+1+\varepsilon^{2}}$ (S9)

The Eq. (S7) can be updated by (S9) as:

$X_{{NDI}^{-}}=\frac{\varepsilon\delta}{\varepsilon\delta+1+\varepsilon^{2}}$ (S10)

Eq. (S10) can be inputted into Eq. (S5) as:

$X_{NDI}=\frac{\varepsilon X_{{NDI}^{-}}}{\delta}=\frac{\varepsilon^{2}}{\varepsilon\delta+1+\varepsilon^{2}}$

The SOC on negative redox electrolyte ($q_{calculated}^{NDI}$) is defined as:

$q_{calculated}^{NDI}:=\frac{X_{{NDI}^{-}}+2X_{{NDI}^{2-}}}{\left[ NDI \right]+\left[ {NDI}^{-} \right]+\left[ {NDI}^{2-} \right]}$

which can be updated by Eq. (S9) and Eq. (S10) as:

$q_{calculated}^{NDI}=\frac{\varepsilon\delta+2}{\varepsilon\delta+1+\varepsilon^{2}}$

**Figure S5.** The examples of the determination of standard electrode potentials for positive (**A** and **B**) and negative (**C** and **D**) electrode systems ($E_{FeCN}$ and $E_{NDI}$, respectively). The smoothed first derivatives of the time dependencies of the voltage on Positive Tank Monitor during charging (100 mA cm^-2^) and discharging (-100 mA cm^-2^) of AORFB (**A** and **B**, respectively); the smoothed first derivatives of the time dependencies of the voltage on NDI electrode (estimated by the subtraction of Monitor Cell voltage from the voltage on Positive Tank Monitor) during charging (100 mA cm^-2^) and discharging (-100 mA cm^-2^) of AORFB (**C** and **D**, respectively).

**Figure S6.** The mapping of time dependencies of electrode potentials by SOC profiles during AORFB operation (20 mA cm^-2^). A and B: charge and discharge profiles for ferrocyanide/ferricyanide positive electrode, respectively (solid curves – experimental time dependencies of voltage on Positive Tank Monitor; dotted curves – the dependence of SOC calculated using Eq. (4) on the potential of ferrocyanide/ferricyanide electrode); **C** and **D**: charge and discharge profiles for NDI negative electrode, respectively (solid curves – time dependencies of negative electrode voltage estimated by the subtraction of Monitor Cell voltage from the voltage on Positive Tank Monitor; dotted curves – the dependence of SOC calculated using Eq. (11) on the potential of NDI electrode ($\delta$=100)).

**Figure S7.** The AORFB performance deterioration on the charge discharge cycling (60 mA cm^-2^).

**Table S1.** The comparison of performance characteristics of reported NDI-based AORFB.

| Negative  electrolyte | Positive  electrolyte | Concentration of active material (M) | Membrane | Current density mA/cm2 | CE  (%) | VE  (%) | EE (%) | Cycling stability | Ref |
| --- | --- | --- | --- | --- | --- | --- | --- | --- | --- |
| Asp-NDI | K_4_[Fe(CN)]_6_ | 0.3 | Nafion 115 | 20 🡪 100 | 98 🡪94 | 82🡪47 | 80🡪45 | 60 mA/cm², 45 cycles  CE ~96–97%;  VE 68→50%;  EE 65→44%;  CU 95→20% | This  work |
| 4A^4+-^NDI | NH_4_I | 1 | Nafion | 5 🡪 50 | 98 🡪~99 | 82🡪~25 | 82🡪~25 | 25 mA/cm^2^, 500 cycles  CE ~100%  VE ~55%  EE ~55% | [1] |
| [ANDI]Cl_2_ | TEMPO | 0.25 | DSV | 40🡪80 | ~100 | - | ~70🡪~35 | 40 mA/cm^2^  more than 1000 cycles  CE ~100%  EE ~72% | [2] |
| [HANDI]Cl_2_ | TEMPO | 0.25 | DSV | - | - | - | - | 40 mA/cm^2^  more than 1000 cycles  CE ~100%  EE ~72% |  |
| Dex-NDI | TEMPO | 0.5 | DSVN | 40 🡪140 | 100 | 83🡪51 | 83🡪51 | 40 mA/cm^2^  1M NDI: CE ~100% | [3] |
| NDI-DMe | FnCl | 0.1 | DSV | 20🡪100 | - | - | ~82 🡪~45 | 40 mA/cm^2^, 400 cycles  EE : 75.5%  CU: 66.7% | [4] |
| NDI-DEtOH | FnCl | 0.1 | DSV | 20🡪100 | - | - | ~82 🡪~45 | 40 mA/cm^2^, 400 cycles  EE: 74.3%  CU: 67.8% |  |
| (CBu)_2_NDI | K_4_Fe(CN)_6_ | 0.1 | Nafion 212 | 20🡪100 | ~100 | 80🡪47 | 80🡪47 | 40 mA/cm^2^, 5070 cycles  CE ~100%  CU: 84.14 % | [5] |
| (SPr)_2_NDI | K_4_Fe(CN)_6_ | 0.1 | Nafion 212 | 10🡪100 | ~100 | 91🡪41 | 91🡪41 | 0.25 M, 20 40 mA/cm^2^, 1000 cycles  CE ~100% |  |
| NDI-C_2_-MzMe | MiAcNH-TEMPO | 0.1 | DSVN | 20 🡪 120 | ~100 | ~80🡪~40 | ~80🡪~40 | 40 mA/cm^2^, 2400 cycles  CE ~100% | [6] |
| NDI-C_3_-MeMz | MiAcNH-TEMPO | 0.5 | DSVN | 20 🡪 140 | ~100 | ~80🡪~50 | ~80🡪~50 | 40 mA/cm^2^, 1800 cycles  CE ~100% |  |
| Asp-NDI | Na_4_[Fe(CN)]_6_ | 0.5 | Fumasep E630 (K) | 5 🡪 20 | - | - | - | 20 mA/cm^2^, 209 cycles  CE ~99.9%  EE ~87.5% | [7] |
|  |  |  | Selemion SX-053DK | 5 🡪 20 | - | - | - | 20 mA/cm^2^, 48 cycles  CE ~99.9%  EE ~91.9% |  |

[1] Singh, Vikram, et al. Controlling π–π interactions of highly soluble naphthalene diimide derivatives for neutral pH aqueous redox flow batteries. *Advanced Materials* 35.13 (2023): 2210859.

[2] M.G. Pan, W. Wang, H.Z. Wang, J. Ma, M.H. Shao, Z. Jin, High-voltage and durable pH-neutral aqueous redox flow batteries based on quaternary ammonium cations functionalized naphthalene diimide and nitroxyl radical systems, Journal of Power Sources 580 (2023). https://doi.org/10.1016/j.jpowsour.2023.233269.

[3] X. Liu, H. Zhang, C. Liu, Z. Wang, X. Zhang, H. Yu, Y. Zhao, M.-J. Li, Y. Li, Y.-L. He, G. He, Commercializable Naphthalene Diimide Anolytes for Neutral Aqueous Organic Redox Flow Batteries, Angewandte Chemie International Edition 63(25) (2024) e202405427. https://doi.org/https://doi.org/10.1002/anie.202405427.

[4] Z.R. Wang, X. Liu, X.R. Zhang, H. Zhang, Y.J. Zhao, Y.W. Li, H.Y. Yu, G. He, Realizing one-step two-electron transfer of naphthalene diimides via a regional charge buffering strategy for aqueous organic redox flow batteries, Materials Horizons 11(5) (2024) 1283-1293. https://doi.org/10.1039/d3mh01485a.

[5] H. Zhang, C.J. Liu, Z.R. Wang, X. Liu, Z.K. Han, X.R. Zhang, Y.W. Li, Q. Zhao, G. He, Synergistic ionic modification strategy enhances the stability of naphthalene diimide zwitterions for cost-effective aqueous organic redox flow batteries, National Science Review 12(5) (2025). https://doi.org/10.1093/nsr/nwaf123.

[6] Zhang, Xuri, et al. Multi-hydrogen bond engineered imidazolium-functionalized naphthalene diimides for stable two-electron storage in aqueous organic flow batteries. *Energy Storage Materials* (2025): 104527.

[7] Mahsa Shahsavan, Cedrik Wiberg, Andrea Hamza, Aapo Poskela, Johan Hjelm, Pekka Peljo, Batteries & Supercaps 2026, 9, e202500764. https://doi.org/10.1002/batt.202500764

**Figure S8.** The recovery of disbalanced AORFB by the of positive tank re-filling by well-balanced electrolyte. Charge discharge current density 60 mA cm^-2^.

**Supporting Note 4.**

**Hydrogen peroxide generation in negative tank**

Off-line UV-vis analysis was utilized for the qualitative assay of produced H_2_O_2_. An aliquot (1 μL, 10 μL and 50 μL) of negative electrode redox electrolyte were collected. Then the excess (299 μL, 290 μL and 250 μL, respectively) of stirred freshly prepared solution of HRP (0.75 ng ml^-1^) and TMB (30 mg ml^-1^) in 0.1 M phosphate-citrate buffer solution pH 6 were added to each of the aliquot. Then the absorbance of the mixture was measured (at 653 nm) by UV-vis plate reader (BioTek Synergy H1 Hybrid Multi-Mode Reader).

|  |  | Composition | Absorbance  a.u. |
| --- | --- | --- | --- |
| 1 | Sample | 10 uL sample + 290 uL peroxide test solution | 0.204 |
| 2 | Sample | 1 uL sample + 299 uL peroxide test solution | 0.091 |
| 3 | Sample | 50 ul sample + 250 uL peroxide test solution | 3.484 |
| 4 | Sample | 300 uL sample | too high, overload |
| 5 | Background | 10 uL sample + 290 uL DI water | 0.152 |
| 6 | Background | 300 uL DI Water | 0.038 |
| 7 | Background | 10 uL sample + 290 uL buffer solution | 0.157 |

The absorbance values of the samples 1-4 is higher than the corresponding backgrounds. This qualitatively illustrates the presence of hydrogen peroxide in the negative tank.

**Figure S9.** Redox processes of NDI before and after AORFB operation. The cyclic voltammograms recorded in three-electrode cell with diluted redox electrolytes (50 mM NDI in 1 M NH_4_Cl, scan rate 20 mV s^-1^; before and after AORFB operation as blue and black curves, respectively) on glassy carbon electrode.

**Supporting Note 5.**

**Analysis of negative electrolyte by HPLC-MS**

Off-line analysis by HPLC-MS showed the clear presence of pristine NDI in samples before and after (Fig. S13A,C and S13B,D, respectively) AORFB operation. The chromatogram of pristine NDI obtained on evaporative light scattering detector (ELSD, Fig. S10B) showed the appearance of the major peak at 0.41 min and minor peak at 0.522 min illustrating the presence of two compounds. The evaluation of the major peak by mass spectroscopy showed the presence of the molecular weights of 499.9 and 497.4 for positive (Fig. S10A) and negative (Fig. S10C) modes, respectively. These values are consistent with a molecular weight of pristine NDI: 498.4 (Inset in Fig. S10B). The presence of minor peak on negative mode with a molecular weight of 995.6 could reflect the presence of anionic dimer of NDI. Similarly, the presence of the minor peak for the molecular weight of 1014.8 visible on positive mode could illustrate the presence of cation dimer of NDI with single ammonia as counter-ion of carboxylic group. Coherently, the major peak at ELSD (0.41 min) showed a UV-vis absorbance spectrum (Fig. S11) typical to NDI [S.M. Wagalgave, S.D. Padghan, M.D. Burud, M.A. Kobaisi, D.D. La, R.S. Bhosale, S.V. Bhosale, S.V. Bhosale, Supramolecular super-helix formation via self-assembly of naphthalene diimide functionalised with bile acid derivatives, Scientific Reports 9(1) (2019) 12825. https://doi.org/10.1038/s41598-019-49235-5].


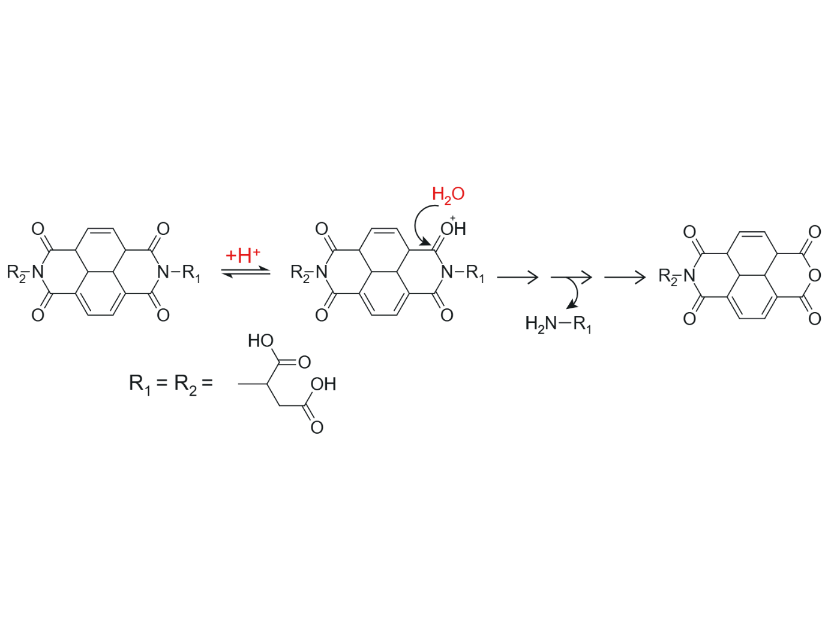

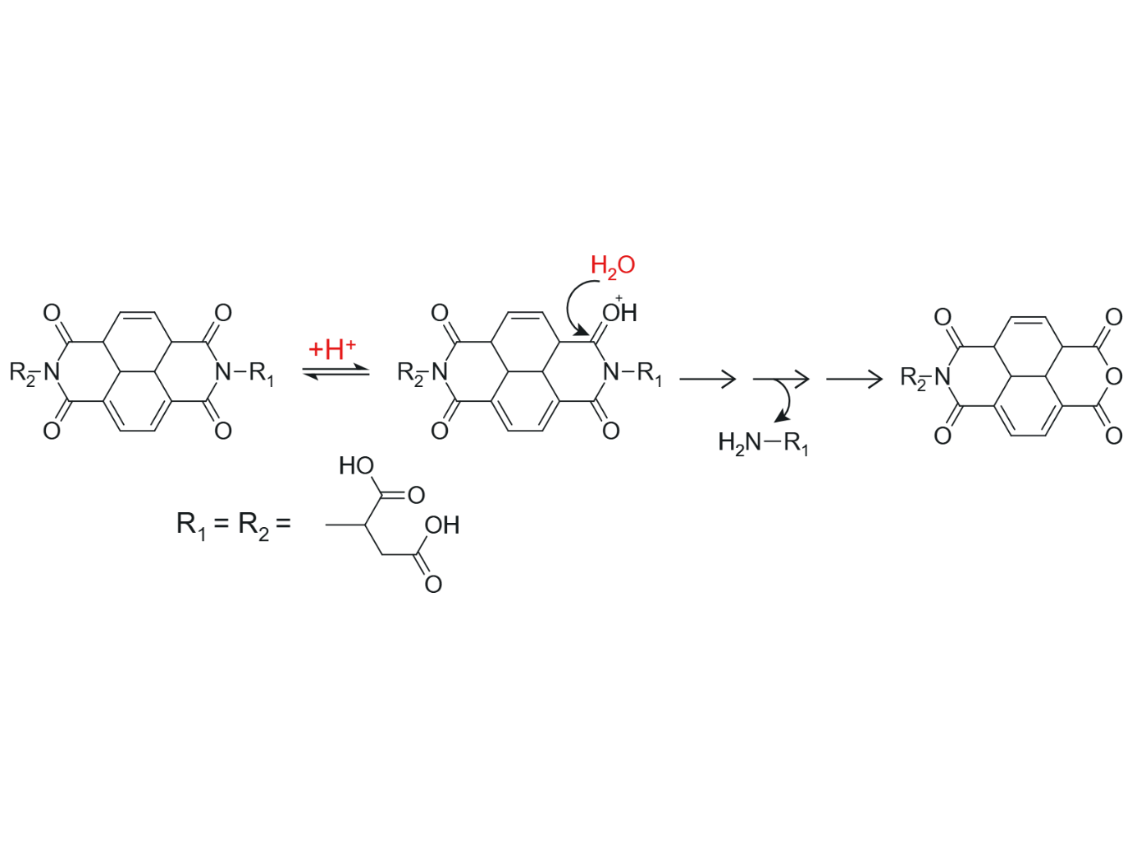


**A, MS+**

**B, ELSD**

**C, MS-**

**Figure S10.** The responses of positive (**A**) and negative (**C**) modes of mass-spectroscopy on the main peak (0.41 min) on chromatogram obtained with ELSD (**B**) obtained for pristine NDI solution.

**Figure S11.** UV-vis absorbance spectra of ELSD main peak (0.41 min), which corresponds to NDI.

The evaluation of the minor peak on ELSD chromatogram (0.522 min, Fig. S12B) by mass spectroscopy showed the presence of the molecular weights of 383.3 and 381.1 for positive (Fig. S12A) and negative (Fig. S12C) modes, respectively. These values are consistent with a molecular weight of NDI monoimide: 383.3 (Inset of Fig. S12B). The presence of monoimide in pristine NDI solution could illustrate the slow hydrolysis on NDI by Gabriel synthesis of primary amines (Fig. 6, Supporting Note 6).


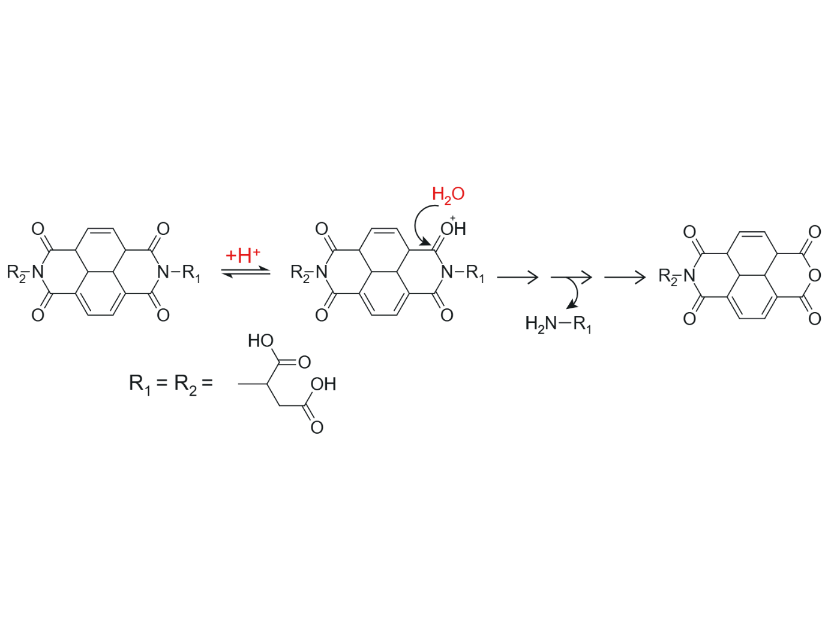

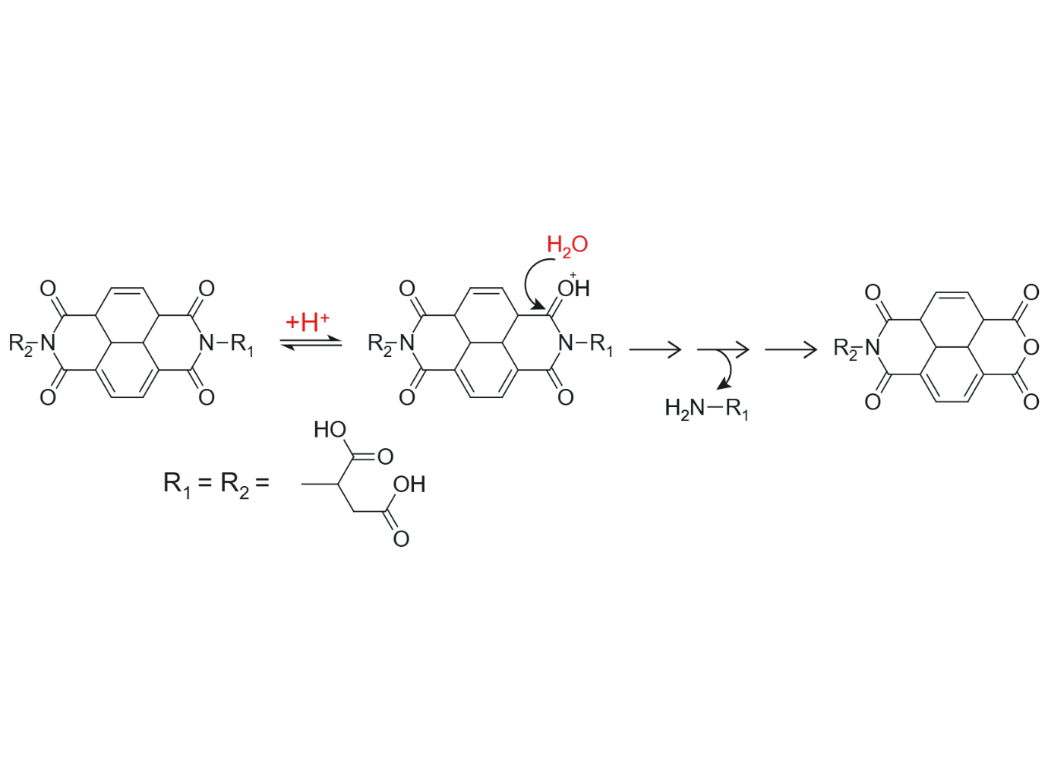


**C, MS-**

**A, MS+**

**B, ELSD**

**Figure S12.** The responses of positive (**A**) and negative (**C**) modes of mass-spectroscopy on the manor peak (0.522 min) on ELSD (**B**) obtained for pristine NDI solution.

The direst comparison of chromatograms obtained on ELSD and on photodiode array (PDA) for NDI solutions showed that the AORFB operation resulted in the charges in NDI solution. Specifically, both the increase of the peak associated with monoimide (0.542 min, Fig. S13B) as well as appearance of the new peaks at 0.6 min and at 0.72 min are visible (Fig. S13B and S13D). In contrast to ELSD (Fig. S13B), PDA showed the poor visibility for the new peak at 0.6 min (Fig. S13D), which implies that associated compound is not aromatic.

The detailed analysis of the new peak appeared at 0.71-0.76 min by negative mode mass-spectroscopy (Fig. S13) showed that it corresponds to aromatics identical to NDI (UV-vis spectra Fig. S14) with a molecular weight of the molecule is 381.1 and 382.2. This implies that the AORFB operation resulted in the increase of the monoimide content visible as appearance of the peak at 0.71-0.76 min in parallel to the peak observed in NDI solution before AORFB operation.

**before**

**Figure S13.** The chromatograms obtained on ELSD (**A**, **B**) and on PDA (**C**, **D**) NDI solution before (**A**, **C**) and after (**B**, **D**) AORFB operation.

**after**

**before**

**after**

**D, PDA**

**C, PDA**

**B, ELSD**

**A, ELSD**

**MS-**

**Figure S14.** The mass spectrum in negative mode of the peak 0.71-0.76 min observed on ELSD (Fig. S13B) and on PDA (Fig. S13D) NDI solution after AORFB operation.

**Figure S15.** UV-vis absorbance spectra of ELSD main peak (0.71-0.76 min), which corresponds to monoimide of NDI.

The detailed analysis of the new peak appeared at around 0.603 min (Fig. S13B) by positive and negative modes mass-spectroscopy (Fig. S16A and S16B, respectively) showed the primary mass weights are within the range 100-250. This could correspond to aspartate liberated due to hydrolysis of NDI.

**A, MS+**

**C, MS-**

**Figure S16.** The mass spectrum in positive (A) and negative mode (B) of the peak 0.6-0.76 min observed on ELSD (Fig. S13B) of NDI solution after AORFB operation.

**Figure S17.** The contamination of NDI solution after AORFB operation by ferrocyanide/ferricyanide. The redox process of ferrocyanide/ferricyanide visible by cyclic voltammograms recorded in three-electrode cell with concentrated negative redox electrolyte (0.3 M NDI in 1 M NH_4_Cl, scan rate 50 mV s^-1^; before and after AORFB operation as blue and black curves, respectively) on glassy carbon electrode as working electrode.

**Supporting Note 6.**


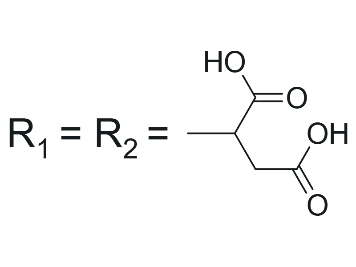

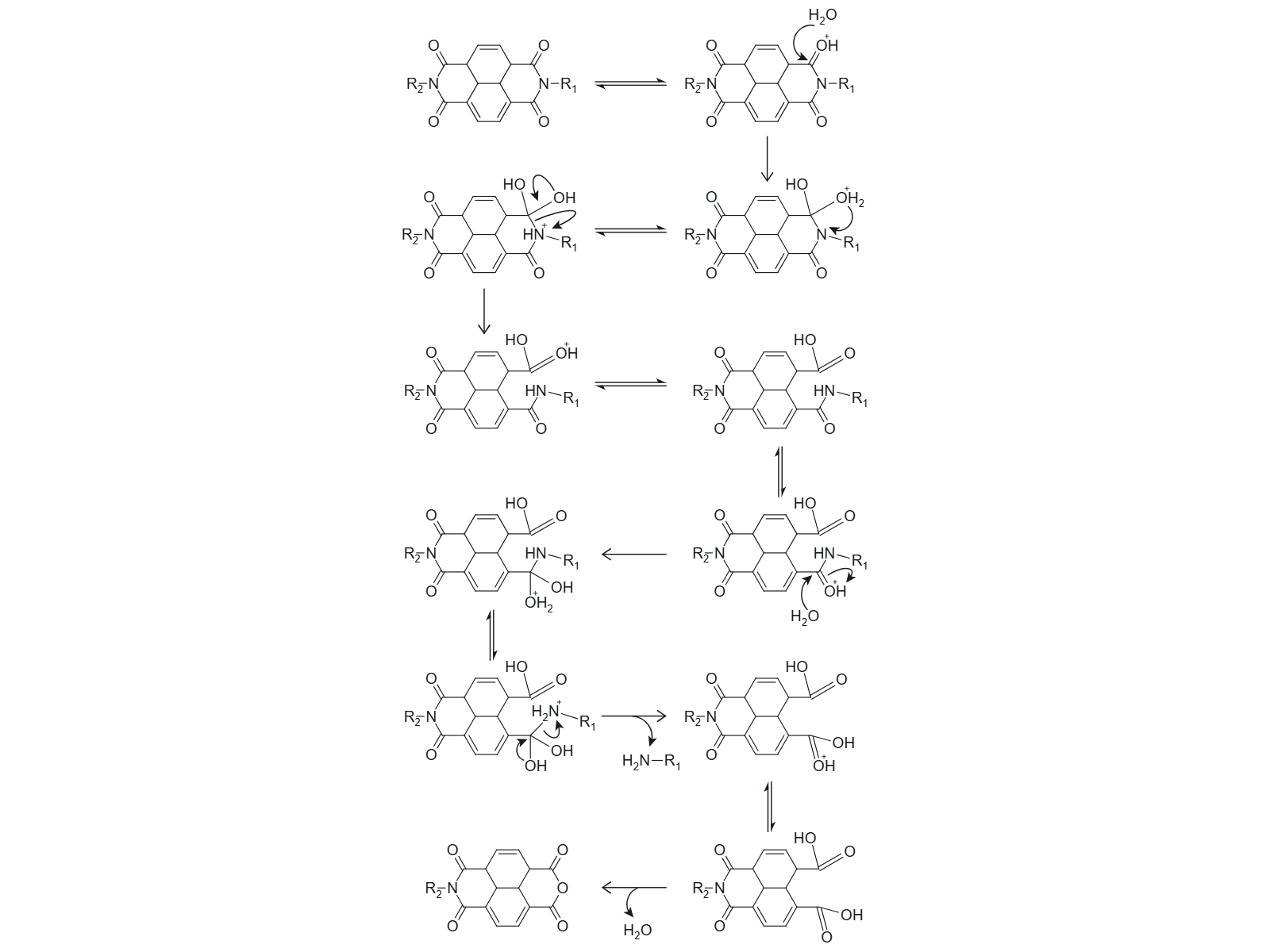
**The mechanism of acidic hydrolysis of NDI**
